# Space and Well-Being in High Security Environments

**DOI:** 10.3389/fpsyt.2022.894520

**Published:** 2022-05-30

**Authors:** Thomas Ross, Jan Bulla, María Isabel Fontao

**Affiliations:** ^1^Forensic Psychiatry and Psychotherapy, Reichenau Psychiatric Center, Reichenau, Germany; ^2^Department of Psychology, University of Konstanz, Konstanz, Germany

**Keywords:** forensic mental health, prison, therapeutic architecture, therapeutic design, narrative synthesis

## Abstract

Research into the spatial dimensions of deprivation of liberty and psychiatric hospitalization has a long and complex tradition. In this context, the increasing numbers of prisoners and patients in forensic hospitals have impressively shown how difficult it is to ensure security, therapy and rehabilitation when space is scarce or not well-suited. In this narrative review, we present the main findings of recent lines of research on spaces in prisons and forensic psychiatric wards, with particular attention to the links between overcrowding in prisons and secure forensic psychiatric hospitals and violence, the foundations of prison and hospital architecture, and on how the design of spaces in prisons and hospitals can influence well-being. We assess and discuss these findings in the context of the current debate on how well-being in secure spaces can support the achievement of rehabilitation goals even in overcrowded institutions.

## Introduction: International Trends on Forensic Mental Health Services in Western Countries

In Europe, there has been a well-documented trend toward increased placement of psychiatric patients who have offended in forensic psychiatric institutions for several decades. This trend has been noted in several Western European countries, and it continues to the present day. There is also evidence of a similar trend outside Europe, e.g., in Canada and the United States. Jansman-Hart et al. (1) reported these developments in an analysis of a number of European countries as well as Canada and the United States, albeit to different degrees. In Canada, the number of new defendants entering the system doubled annually from 1992 to 2004. In the United States, a related development called the “forensification” of state hospitals was reported. Between 1999 and 2014, responses to a nationwide survey on the number of forensic patients in state psychiatric hospitals showed a 76% increase in the number of forensic patients nationwide. The largest increase was in people who were court-ordered after being found incompetent to stand trial and requiring inpatient recovery services (2). Several countries in Western Europe (Austria, Denmark, England, Germany, Ireland, Italy, Netherlands, Spain and Switzerland) reported an increase in the number of forensic beds by an average of 110% between 1990 and 2006 (1). (3, 4) reported on nine European countries with regard to their use of forensic psychiatric services and described for 1990, 2002 and 2006 a clear increasing trend in all these countries. Around the same time as this work, Salize and Dressing (5) studied the admission of mentally disordered offenders to specialized forensic care in fifteen states of the European Union. These results all point in the same direction, and it can be assumed that this trend has continued over the past and current decade in many European countries, although not all [e.g., Czechia (6), or Finland (7)]. For England, however, increasing occupancy has been safely documented up to 2016 (8), with Keown et al. (9) linking the development of general psychiatric beds to transfers from prison to forensic psychiatric hospitals. The closure of mental health beds was accompanied by an increase in referrals from prison to hospital for treatment (civil or forensic involuntary detentions). Convincing evidence of long-term significant increases in placements in forensic psychiatric facilities is also available from Germany (10–13).

The physical environment has been described as one of the central determinants of mental health and well-being (14), and the importance of a comprehensive understanding of the concepts of space and place for mental health and care has been stressed by researchers of different disciplines (15). Due to the coercive nature of secure forensic environments and the space restrictions they impose on residents, an increased potential impact of the physical environment on their well-being can be assumed. In a report from 2012, the United States Government Accountability Office stated that overcrowding “affects inmates’ daily living conditions, participation in programs, meaningful work opportunities, and visitation,” including “congregating inmates at higher risk of violence and more potential victims for longer periods of time,” “inmate waiting lists [for programs] and idleness,” the inability to “meet inmates’ substance abuse or educational needs,” “fewer opportunities to do meaningful work,” “crowded visiting rooms [that] make it difficult for inmates to visit their families,” and increased “staff stress and overtime” and “fewer correctional staff on board than needed” [(16), pp. 18–23]. It also explicitly warned that prison overcrowding could increase the risk of violence, especially in high-security facilities that house the most dangerous prisoners, and compromise the achievement of rehabilitation aims.

Previous review studies on similar topics reported findings on architecture and design in prison services (17) and described clinical, legal and structural aspects of forensic hospital design (18). In this paper, we add on the scope of previous work: First, we provide an overview of research on the effects of overcrowding in custodial institutions and hospitals. In the next section, theoretical models and selected findings on the relationships between physical environment and well-being in non-forensic settings are presented. We then move on to specific results of research in high security environments which add to the general findings on space in therapeutic spaces and design. Finally, we discuss the possibility of applying these notions to enhance well-being in stressed secure forensic spaces.

The relevance of the topic arises in view of rising numbers of prisoners and patients in forensic hospitals (and related phenomena, e.g., compulsory psychiatric admissions), which can lead to overcrowding in custodial institutions and require facility extensions or new buildings. There is a wide variation in the provision of secure forensic mental healthcare across international jurisdictions; in this paper, the term “high security environments” refers to forensic facilities of different levels of security (e.g., low, medium, high) and includes both custodial and hospital environments. The notion of “well-being” includes biological, psychological, and social dimensions (19). Generally, we focus on well-being as it relates to a (sense of) safety and to the absence of clinical psychopathology resp. of stressors (e.g., violence and aggression, self-harm and suicide) which negatively affect mental health.

## Methods

A literature search was carried out between January and February 2022 in the databases PsycInfo, PSYNDEX, and Google Scholar. The search terms were: overcrowding (or) crowding (or) overcrowded (or) density (or) bed occupan*; architecture (or) architectural design (or) building design; prison (or) jail or incarceration (or) imprisonment (or) correction facilities; forensic psychiatry (or) forensic mental health (or) secure unit (or) forensic care. A preliminary selection was made by two authors independently. Second, the reference lists of the publications that emerged in the preliminary selection were searched for potentially relevant literature. The final selection was made by two authors according to the relevance for the main topics of the narrative review. The included publications were published in English language in a scientific journal between 2000 and 2022; one book chapter and one doctoral dissertation were also included. Because of the scope of the review, which also covers theoretical models and interdisciplinary approaches, different types of publications were included: original research, review papers, and papers with a focus on theory or methods.

## Results

The majority of the 31 included publications come from English speaking countries (United States: 7: United Kingdom: 7; Australia: 5; Canada: 1); four publications come from Sweden, one from Finland, two from Switzerland, one from Germany, and one from Turkey. There were 16 original studies, ten reviews and five publications with a focus on theories/methods. Thirteen publications investigated the prison setting including one pre-trial detention center), six publications focused on forensic hospitals; in-patient psychiatric wards, mental health facilities, and therapy rooms were also investigated (view Supplementary Tables 1–3).

### Hospital Overcrowding

Overcrowding in hospitals often leads to safety concerns among mental health professionals and hospital managers. The greatest risk is seen in an increase in aggressive and violent attacks by patients on fellow patients and staff. The evidence for a significant association between overcrowding and aggressive patient behavior is mixed and often limited to small studies. Virtanen et al. (20) investigated the association between ward overcrowding and violent physical assaults in inpatient psychiatric acute hospital wards in Finland. Staff members (*n* = 1098) were asked to report the timing of physical assaults on themselves and on ward property. Almost half of the hospital staff worked in overcrowded wards, and an overcrowding rate of more than 10 per cent units at the time of the event was associated with violent assaults on staff [odds ratio (OR) = 1.72, 95% CI 1.05–2.80; OR = 3.04, 95% CI 1.51–6.13 in adult wards], after adjustment for confounding factors [age and gender; type of occupation (physician/head nurse/registered nurse/licensed practical psychiatric nurse), type and length of job contract, hospital district and specialty, sum of patient days per month]. No association was found with attacks on ward property (OR = 1.06, 95% CI 0.75–1.50). The study has been criticized in light of methodological issues related to criteria of causation, which may not all have been fulfilled before a causal relationship between higher occupancy rates and violence can be established. Kapoor (21) argued that in addition to the reported dose-response relationship, uncontrolled staffing variables and ward acuity levels could also be associated with the occurrence of aggression. Despite this criticism, the Virtanen, Vahtera et al. study is still considered important evidence of an association between overcrowding and assaults against staff on psychiatric wards. Weltens et al. (22) presented an extensive systematic literature review on the risk factors for aggression and violent assaults on (general) psychiatric wards. The explanatory factors for aggressive behavior were divided into patient, staff and ward factors. Significant risk factors on the wards were higher bed occupancy, busy places on the ward, walking rounds, unsafe environment, restrictive environment, lack of daily structure, smoking and lack of privacy. Most of the studies included in this literature review indicated a substantial positive association between overcrowding and aggression, but one study found no correlation between the number of patients in the ward and aggressive incidents. Referring to this, in the subsection on bed occupancy, the authors state that “the evidence for overcrowding as a factor contributing to the occurrence of aggression is contradictory, but there is some evidence that overcrowding is associated with the occurrence of aggression” [(22), p. 16].

### Prison Overcrowding

Prison overcrowding has long been defined in the literature as a safety or health problem. As early as the late 1970s, the occupancy rate in United States prisons began to rise sharply. In the 30 years from 1975 to 2005, the number of people in prison increased sevenfold. The United States imprisons far more people per 100,000 resident population than any other country in the world for which relevant data is available. For the years 2003 and 2004, the number of incarcerated persons was 726 persons per 100,000 inhabitants; by comparison, Canada had 116, Italy 98, Germany 96, France 91, Sweden and Switzerland 81 during the same period [(23), pp. 416–17].

In view of the development in the United States, the problem of overcrowding and its potential impact on safety and health became the focus of scientific research in other countries as well. There is hardly anyone who does not see overcrowding in prisons as a problem. Massive overcrowding, such as that which existed in the southern states of the United States, places a considerable burden on prisoners, but also on correctional officers, who face very high levels of stress and threat (24). Baggio et al. (25) investigated the relationship between prison violence and institutional factors in a Swiss remand prison between 2013 and 2018, measuring violence (assaults requiring immediate medical treatment) as well as annual overcrowding and turnover rates. The results showed that prison violence was higher when overcrowding and turnover increased. In a large study on prison structure, inmate mortality and suicide risk in Europe by Rabe (26), the author found an increased risk of suicide for sex offenders, violent offenders and prisoners sentenced to short and long-term imprisonment. In addition, prison mortality was associated with overcrowding.

However, the correlations are not always as clear-cut as they may seem based on these studies. In fact, there is mixed evidence on the relationship between prison violence and institutional factors such as overcrowding and turnover, and recent research suggests that these factors may not be important or relevant. For a prison population in Switzerland, Wolff et al. (27) showed that overcrowding of more than 200 per cent was associated with self-strangulation or hanging, but not with all self-harm cases. A meta-analysis by Franklin et al. (28) showed that overcrowding in prisons is often not always associated with aggressive assaults or security-related restraints. Looking at their findings, the authors summarized that prison overcrowding has little substantive impact on inmate misbehavior. However, the evidence for the negative effects of prison overcrowding in general is overwhelming. Walker et al. (29) conducted a systematic literature review on changes in prisoners’ mental health and examined how these relate to various aspects of incarceration and the prison environment, including overcrowding. From fifteen longitudinal studies included in the review, the authors concluded that isolation, overcrowding and larger prisons are associated with poorer mental health in prisoners, which is likely to place a particular burden on prison staff and mental health services in addition to the problems experienced by prisoners.

Similar caution must be exercised in assessing the suicide risk of incarcerated persons. In general, the suicide risk of prisoners is significantly higher than that of the general population in many countries [(30), figure 1, p. 950]. However, a whole range of factors play a role here, so that the higher risk cannot be unequivocally attributed to overcrowding. van Ginneken et al. (31) conducted a study on suicide rates as a function of occupancy rates based on data from the Ministry of Justice for adult prisons in England and Wales (2000–2014). Larger population size, higher prison turnover, higher security and public management were associated with higher suicide rates. However, when controlling for these factors, overcrowding was not related to suicide rates. Fazel et al. (30) studied 3,900 prison suicides from 24 countries between 2011 and 2014 and tested associations between suicide rates and 11 factors related to prisons and health services, including overcrowding. Again, there was no significant relationship of overcrowding with suicide risk.

In sum, empirical evidence to date is not conclusive about the effects of overcrowding in prison on violence against others or self-harm. On the other hand, the continuous efforts of researchers to study these effects may reflect the perception of prison administrations and stakeholders that overcrowding is a pervasive phenomenon that can affect the foundations (i.e., security) and rehabilitation goals of the prison system.

### Therapeutic Architecture and Design: Models and Findings in Non-forensic Settings

There is no doubt that the physical environment affects building users, and there has been considerable debate about the relevant subtopics and the most suitable methodologies, e.g., interdisciplinary research, evidence-based design, or occupants’ feedback/users’ needs (32). Post-occupancy evaluations (POE) is a diagnostic and research tool that includes “the process of examining and evaluating the functioning of a building in a systematic way after completion” [(33), p. 58]. Typically, the researchers gather feedback from users in form of social and behavioral data and seek to establish a link between building design and use behaviors. This approach generated a considerable amount of knowledge in the field. However, criticism was raised: over the long term, the emphasis on measuring users’ satisfaction could be less effective for building design. Instead, a shift to evidence-based design was called for. Similar to evidence-based medicine, this approach assumes that the results of research (POE or other feedback studies, field research in sites or buildings, laboratory research) are necessary to make good design and building decisions possible (34).

An optimal treatment environment for mentally ill people places special demands on the architecture and furnishing of buildings and interior spaces (35). The term “healthcare architecture” is often used in this context, and significant progress has been made in this field in many western industrialized countries in recent decades. Deinstitutionalization and community-based treatments are some of the main attempts that have been made to create a more “normal environment” for those affected.

The “Normalization Theory” was introduced into mental health care from the adjoining field of learning disabilities (36). It states that institutions for mentally ill people should have as many references as possible to a normalized interior design in order to mitigate institutionalization and support reintegration and rehabilitation for patients in closed institutions (17). However, it is clear that psychiatric care cannot be completely deinstitutionalized anywhere. In addition to a living environment that should be kept as close to everyday living as possible, larger inpatient facilities require a therapeutic environment with appropriate spaces which support the therapeutic work. Because this cannot be achieved with the architecture of the family home, Chrysikou (37) called for a “Fit for Purpose” architecture for the mentally ill and proposed a theoretical model on the basis of a modern therapeutic architecture which can be implemented. The empirically based SCP model was developed according to three parameters that define mental illness: safety and security, competence, and personalization and choice, and it takes into account the main requirements or needs of mental health care. Regarding the first parameter, safety and security, risks include harm and self-harm, violence and abuse, vulnerability, substance abuse, self-neglect and noise. Competence refers to the ability of clients to maintain a certain level of independence to take care of themselves both physically and socially, with the ability to live independently being the optimum. Personalization and freedom of choice, the third parameter, refers to the degree of freedom the client can achieve in a facility (37). A recent rapid review by Oostermeijer et al. (38) found preliminary evidence that the use of good design and architecture principles can help to prevent measures of seclusion and restraint in psychiatric facilities. The importance of the physical environment in psychiatric settings has also been considered in a safety model of psychiatric care, e.g., the Safewards model (39). This model aims to reduce risk and coercion on inpatient wards and postulates six areas that influence conflict and containment rates: the patient community, patient characteristics, the legal framework, the staff team, the physical environment and the environment outside the hospital. The physical environment includes, among other things, the quality and complexity of the buildings, with high quality and comfort eliciting greater care and respectful interaction with patients, as well as the presence of isolation rooms and locked doors or psychiatric intensive care units. In turn, staff interact with the physical environment by, for example, ensuring that the building is well maintained or adapting it to patients’ preferences. According to a recent study, the Safewards model has shown a positive overall impact on the frequency of conflicts and the containment of problems in psychiatric units (40). However, it was not reported whether there was a specific effect of the physical environment.

In a comprehensive review on mental health and architecture, Connellan et al. (32) identified 13 (partially overlapping) topics on the effects of the architectural designs of mental health facilities on the users, including security/privacy, light, gardens, impact of architecture on mental health outcomes, interior design, psychogeriatric, and forensic psychiatric facilities. The authors concluded that there is a shortage of space and a lack of privacy in wards. Moreover, violence against patients and staff is widely prevalent and increasing. They emphasized the importance of providing demarcated spaces for particular activities and, in general, more space.

### Therapy Rooms and Outdoor Therapy

The characteristics of spaces in which therapies are conducted could improve the psychotherapeutic process and patient well-being. Sinclair (41) explored the views and experiences of 24 clients and 21 therapists on the physical environment of the therapy room for counseling and psychotherapy. Comfortable seating and a comfortable room temperature, soundproofing, no interruptions and accessibility of the room were ranked as most important by clients and therapists. Participants reported that feeling physically comfortable and safe in a room enabled greater engagement in the therapeutic process. Rooms with a “clinical” appearance were described as not helpful. Three main themes emerged from the responses: Comfort, the appearance of the room and the room as a workspace. It is likely that these themes can also be applied to institutionalized contexts where psychotherapy takes place [see also (42), for a similar small-scale study]. Waiting rooms may affect mental states (emotions, expectations, memories, etc.) prior to the therapeutic session, and the application of principles of therapeutic design can increase the benefits of the therapeutic intervention (43).

A larger study by Backhaus (44) also investigated clients’ and therapists’ perceptions of the importance of the physical environment of the therapy room. Specifically, Backhaus wanted to find out what importance clients and therapists attached to accessories, colors, room design, furniture, lighting, temperature, and sounds. In addition, the study examined the connection between client loyalty and the physical environment of the therapy room. The sample consisted of 226 participants (73 therapists and 153 clients). The physical environment had a significant impact on the ability of clients and therapists to establish a therapeutic relationship. Accessories and colors were rated as least important, while sound was rated as the most important attribute of the therapy room. In addition, room design was rated as more important than furniture and lighting. Therapists rated lighting as more important than accessories and furnishings, and clients rated furnishings as more important than lighting and accessories. The results also showed that lighting was significantly correlated with clients’ perceived competence and trustworthiness of the therapist. Although the research topic of architecture and design in therapeutic rooms has received increased attention in the last decades, it is unclear to what extent clinical practitioners are aware of these relevant findings and recommendations (43).

Talking therapies in the outdoors can also have physiological and psychological benefits, such as reduced stress responses and improved mood for both patients and therapists. With the aim of providing a framework for best practice, Cooley et al. (45) published a review of the outdoor experiences of therapists and their clients (38 articles published between 1994 and 2019 containing data from 322 therapists and 163 clients). The outdoor context for therapy ranged from sitting or walking in urban parks and forests to remote expeditions into the wilderness. The bottom line was that patient and therapist well-being improved with positive effects on relationship building and stabilization.

### Architecture and Design in High Security Environments

#### Prisons

The architecture and design of buildings and living and therapy spaces in specially screened settings must now meet a number of conditions in order to achieve modern standards. Particularly in the United States and in other western countries where prisons are overcrowded, the design or redesign of prisons is seen as a way of legitimizing the execution of sentences by making these as humane as possible, so that prisoners and staff experience justice as fair or balanced. According to St John (46), the core idea of “placial justice” is that social justice can be improved through open, transparent and inclusive (OTI) design in prison buildings. Assuming that this approach can strengthen the penal legitimacy and rehabilitative function of the prison system, the author posits that the principles of OTI design should be taken into account especially in the planning of new buildings or extensions of prisons. Grant and Jewkes (47) outlined the history of prisons in the United States and Australia and argued that for a long time Australia uncritically imitated the penitentiary concepts of the United States. According to the authors, this led to prison design and policies that were not well-suited to the local conditions, resulting in hard-to-manage prisons, poor staff morale and a significant number of non-violent and violent incidents related to poorly designed environments. The 21st. century saw the emergence of genuine Australian approaches to prison design that better addressed the needs of particular groups of prisoners. A number of prisons have been built to provide therapeutic, drug-free and treatment environments for HIV-positive and mentally disabled prisoners, for prisoners with substance abuse problems, women, sex offenders or special facilities for Aboriginal people [(47), p. 238]. They concluded that “(t)he prevailing trend is away from traditional cell design toward cottage-style communities, i.e., accommodation units laid out on a campus,” and progressive design can be paired with secure cell technology [(47), p. 239].

In 2018, the Research and Evaluation Unit of the Swedish Prison and Probation Service (SPPS) conducted a rapid review on behalf of the Real State of the SPPS. The aim of the review was to embed research findings on good architecture and design in policy discussions about large-scale, long-term investment decisions. The review summarizes the academic research on the role of physical environment in prison services. The authors included nine previous reviews on the significance of architecture and design in institutional settings and extended the findings with recent literature (18 journal articles, nine book chapters, and one monograph). The main findings were that a normalized or homely environment and reduced overcrowding (by international standards) contribute to higher levels of well-being for both inmates and staff. Room layout is beneficial as it provides a clear overview and offers natural opportunities for staff and inmates to meet. From the previous reviews, the authors deduced that natural light or lighting similar to daylight, as well as access to nature, can reduce stress and increase well-being. In addition, prisoners may be more affected than other groups by poor physical environments such as overcrowding, noise and inadequate ventilation. Focusing on the Swedish prison system, the authors noted that more could also be done to build centralized knowledge about the current state of Swedish prison facilities from a rehabilitative architecture and design perspective within the agency. They also saw room for improvement in the implementation of supportive environments, as well as in the application of best practices in new facility expansion [(17), p. 5].

Until a few years ago, there was little research on the factors that influence staff-prisoner relationships, and prison building design was not considered either. However, relationships between correctional officers and prisoners are crucial to prison life and affect prison order and the well-being of prisoners and staff. Beijersbergen et al. (48) investigated the relationship between prison architecture and prisoners’ perceived relationship with officers using data from a large Dutch prison project with over 1,700 prisoners in 32 Dutch pre-trial detention centers. Prison design was related to the relationship between officers and prisoners. Prisoners in panopticon prisons in particular were less positive about officer-prisoner interactions than prisoners in other facilities. Older units and units with many double cells also had a detrimental effect on officer-prisoner relations. Morris and Worrall (49) examined the relationship between two types of prison architecture (telephone pole design and campus design) and misconduct of male prisoners in Texas. The data was collected from 2,500 inmates, and inmate-level and prison-level predictors were included in the multilevel analyses. The results suggest that prison architecture was associated with non-violent misconduct (being assigned to a campus-style unit increased the odds of a property infraction, and of a security-related infraction), but not with inmate violence, drug-related misconduct, or reported possession of contraband. As for the limitations of the Morris and Worrall study, the authors acknowledged the use of a simplistic measure of prison architecture (aerial photographs), suggesting that measuring the characteristics of prison architecture requires more complex methods.

Satisfaction with the physical work environment can have positive effects on work efficiency and job commitment, and reduce staff turnover. Conversely, harsh work environment conditions in prison (intrusive noise, clutter, shabbiness) were related to decreased well-being, measured as somatic and psychological symptomatology, increased use of alcohol and tobacco, and sick leave rates (17).

#### Forensic Hospitals

The review of Connellan et al. (32) identified two research papers on the effects of physical environments in forensic hospitals. The central topics were architectural design, safety, privacy, and escapes. Similarities between those papers included a comfortable domestic scale and ambience, security, a pleasant, domestic atmosphere that is well-lit by natural light, and ample space for the patients. These topics were also focused on in later studies, adding on the findings on prison architecture and design presented above.

Three recent studies from Sweden were aimed at evaluating the effects of the relocation in new purpose-built, evidence-based forensic psychiatric facilities by collecting patient and staff feedback in three different forensic hospitals. Olausson et al. (50) investigated the patients’ experiences of place and space using a qualitative design and thematic content analysis. Four themes emerged from the data: a private place, maintaining self-esteem, feeling comfortable and harmonious, and relating to one’s life. From this, the authors concluded that purpose-designed environments can support daily life and well-being and create comfort, which is seen as very therapeutic by patients. Wijk H, et. al. (51) focused on patients’ assessments of the atmosphere on the wards and the quality of care. Baseline data were collected in the old facilities and at three follow-up visits after the move over 3 years. Only seven of the 74 patients initially approached for participation were still present at the third follow-up, but these patients indicated that the quality of care had improved with the move to a new building. These methodological shortcomings illustrate the difficulties of conducting prospective longitudinal research in forensic hospitals, which is crucial to shed light on the long-term effects of the physical environment. The related study by Degl’Innocenti et al. (52) examined staff job satisfaction and perceptions of a person-centered physical and psychosocial environment following a move to a new setting. The results suggest that staff perceptions of ward atmosphere in forensic psychiatric hospitals depend on physical and psychosocial environment factors. Perceived ward atmosphere was assessed by using an instrument to evaluate the extent to which staff felt they were able to provide perception-centered care and the extent to which environmental factors supported them in their work. The move to the new, evidence-based facilities had a positive impact on staff perceptions of the ward atmosphere. Their perception of a person-centered physical and psychosocial environment increased after the move. In particular, they described a greater sense of security and “feeling at home” [(52), p. 28], as well as a greater ability to interact socially in the new work environment.

Eggert et al. (53) investigated related questions with a quasi-experimental study design. They examined the person-environment interaction effects of environmental design on ward climate, safety, job satisfaction and treatment outcomes in a new, high-security forensic psychiatric facility in Colorado. Participants included 879 individuals (*n* = 353 staff and *n* = 526 patients in three security groups) who were interviewed over three data collection periods: 6 months before moving to the new building, 6 months after moving to the new building, and 12 months after moving to the new building (combined experimental group). Staff and patients of the control group (*n* = 378) remained in their previous facilities. No significant differences were found in patient-to-patient and patient-to-staff assaults across the different time periods. For staff, no significant effects were found for any of the three factors of the Copenhagen Burnout Inventory (CBI). Ward climate, operationalized by therapeutic hold, patients’ cohesion and mutual support, and experienced safety (measured with the EssenCES) were not significantly related to personal burnout, work-related burnout and client-related burnout. In sum, the effects of the new environmental design were less than expected and the authors conclude that “for in-patient forensic psychiatric treatment, the facility itself is a tool that requires interpersonal and organizational effectiveness to maximize its potential” (p. 537).

Not only atmospheric impressions are related to environmental variables. The physical environment is purported to influence aggression (see above), and also the use of coercive measures. Van der Schaaf et al. (54) investigated the design features of 199 psychiatric and forensic wards and their relationships with the use of coercive measures on over 23,000 admissions of around 15,000 patients. The 115 design-related variables covered features associated with the quality and the safety of the physical environment and the well-being of patients (privacy; daylight, views and nature; comfort and control; facility level; safety; rooms for seclusion). The authors found 14 design features that significantly affected the risk of being secluded during admission: the presence of an outdoor area, special security measures such as the presence of locking devices on doors with delayed alarm and opening, door position monitoring and a large number of patients in the building increased the risk of being secluded. Protective design features reducing the risk of being secluded were related to the privacy and autonomy of patients and included more private space per patient, a higher level of comfort and better visibility on the ward. The finding of the presence of an outdoor space or garden as a risk of being secluded, which is not consistent with prior studies, can be explained by the use of limited measures. Visibility on the ward can negatively affect the ward atmosphere, but it can also increase the sense of security for patients and staff. These results underscore the complexity of measuring the characteristics of the physical environment as they relate to each other and their subjective impact on users.

Based on a review of the literature on features that make forensic psychiatric facilities best suited to the needs of forensic patients and staff, Seppänen et al. (18) proposed a systematic approach to the complex challenges of designing modern forensic hospitals. Key design issues that need to be addressed include carefully defining the patients to be cared for in the facility, defining the role and profile of the facility in the overall organization of forensic services, and weighing options based on current (pre-existing) facilities: Can the existing facilities be renovated/improved? If not, what are the arguments for the location (urban or rural facilities?). When building new facilities, a number of policy aspects need to be considered (regional, national, financial), and in terms of functional content, therapeutic and safety requirements should be carefully combined to meet the needs of patients and staff. Under the heading “Growth and Change,” the authors listed some of the aspects that need to be considered to ensure that the design is long-lasting and does not become obsolete too quickly [(18), pp. 7–9]. For therapeutic non-forensic settings, theoretical models for contemporary architecture and design have been proposed. These build on the principles of Normalization Theory, but go beyond it by putting emphasis on three key therapeutic factors: safety, competence, and personalization (37). The question arises on as to what extent these models can be applied in high security contexts. Personalization and freedom of choice are still limited in institutions, especially in forensic psychiatric units. Staffing and training, stigma, resources and design can influence the client’s interaction with the facility. Privacy and territoriality, as well as clients’ opportunities to interact with other clients, staff or even people from the community inside or outside the facility, are features of personalization and freedom of choice. With the elements outlined above, this model can be easily transferred to environments where individuals who have committed crimes are to be resocialized and/or treated, i.e., prisons and forensic psychiatric institutions.

But here, too, the correlations between staffing, space availability, increased comfort and facilities, and exterior views of the environment cannot always be reproduced. In a multicentric study with cross-sectional design, Rogerson et al. (55) examined the relationship between the physical design of mental health facilities and the incidence of aggressive behavior in a nationwide study in the United Kingdom that included 101 forensic and non-forensic inpatient wards in seven NHS trusts. The physical environment/architecture of the wards and their general characteristics were assessed by means of the Ward Features Checklist (WFC). A higher score on the WFC dimension 1 “Staffing and space” represented “fewer beds; higher staff to service user ratios on day and night shifts; more dayroom and bedroom space per service user; and more toilets per service user”; a higher score on the WFC dimension 2 “Comfort and facility” included “higher indoor temperature; lower noise levels; fewer rooms open to service users in the day; the opportunity to participate in games with other service users; access to occupational therapy; type of flooring; and ward currently below service user capacity” (p. 3). Clinical ward staff in the participating wards (*n* = 191, estimated 10.3% response rate) completed an online survey focusing on subjective perceptions of safety in the workplace. For the measurement of the outcome variable (incidents of verbal and physical aggression, and property damage), official records for incidents in the prior 6 months were used. Physical aggression was associated with greater staffing and space (dimension 1 of the WFC; incident ratio = 2.19), as well as greater comfort and better facilities and outdoor views of the urban environment (dimension 2 of the WFC: Incident ratio = 1.24). For the verbal aggression and property damage incidents similar results were found as for physical aggression. These findings are not in line with the literature reporting an association of high bed occupation with aggression and coercive measures, but add to research pointing at a link between staffing and incidents (56). The authors acknowledge that there may be “complex organizational and relational factors that need further research to fully understand the overall context” (p. 1). In this sample, forensic wards reported lower levels of physical and verbal aggression compared to acute (non-forensic) wards. These findings open an array of possible interpretations (e.g., reporting culture in wards, attribution to resources according to perceived risks) which are worth investigating.

## Discussion

### Limitations and Strengths

This review is narrative and not strictly systematic. There may have been small-scale international publications in other languages than English that have not been covered in this review. The results are not necessarily generalizable to all countries and settings owing to wide variations in the provision of forensic mental health and custodial care across international jurisdictions. A major strength is the comprehensive character of this review, which outlines the findings on negative effects of overcrowding in both hospitals and prisons. It also shows how architecture and design can influence well-being in hospital and secure forensic settings. Moreover, interdisciplinary theoretical models and methods are presented which can help to understand the complex relationships between well-being and physical environment and how “good spaces” can contribute to healing and rehabilitation processes. In addition to scientists, the results will be of particular interest to (forensic) mental health services and political decision-makers who organize and finance construction projects in the areas addressed here due to the overcrowding situation.

### Synthesis and Conclusion

In some western countries, prisons and forensic psychiatric facilities are overcrowded. Overcrowding creates significant challenges in the management of prisoners and patients. Crowding or lack of space has been linked to additional mental health problems and well-being issues, but a significant correlation between overcrowding and higher rates of aggression and violence cannot be inferred beyond doubt (view section “Results” for details). However, it can be expected that occupancy rates of more than 10% above the structural or organizational maximum increase health risks in prisons and on psychiatric wards. This applies to prisoners, patients as well as staff (20). One way to counter overcrowding is to expand or build new facilities. In recent years, considerable efforts have been made at the international level, both in the correctional system and for psychiatric and forensic-psychiatric hospitals, to create adequate and modern building conditions for the respective requirements of correctional and psychiatric treatment. In the meantime, there are numerous scientifically sound proposals on how rooms can and should be designed in order to achieve their respective purpose (living, therapy, leisure, rest and relaxation) from a therapeutic or a rehabilitation point of view (section “Therapy Rooms and Outdoor Therapy”). To date, there is considerable evidence that architecture and design of prisons and forensic psychiatric facilities are significantly related to measures of mental health, well-being and safety of prisoners, patients and staff (section “Architecture and Design in High Security Environments”). Recommendations for contemporary therapeutically oriented and evidence-based architecture are now available for decision-makers, and these should be taken into consideration when planning extensions or rearrangements of facilities hosting mentally ill and/or high risk populations [e.g., (43)].

The relevant publications on architecture and design in secure forensic environments come from Sweden, Netherlands, and English speaking countries (sections “Prisons” and “Forensic Hospitals”). Relevant international publications from German-speaking or Eastern countries are rare. In view of the very tense occupancy situation in the German forensic system and the construction activity taking place there, it would be desirable to scientifically accompany and evaluate this process on the basis of international experience and findings, and thus contribute to the international development in this field.

When starting new studies, detailed preliminary considerations should be made about the methodological design of the study. A number of different methodological approaches have been used in international research to date (e.g., multisite studies, one site study; cross-sectional, prospective longitudinal; quasi-experimental, qualitative; regression analysis, principal component analysis, multilevel model). In order to capture the effects of and the interaction with the physical environment at different levels (users/patients/inmates; staff/therapists/nurses; institution), several approaches are needed (multilevel/multimethod, etc.), but all of them must be precisely adapted to the conditions of the respective (research) environment in which they take place. The international development in this field has shown how important flexibility is in the selection of suitable methodological approaches for the respective scientific questions [Australia is an impressive example of this, cf. (47)]. In our view, it would not be expedient to limit the relevant research to a generally valid standard, especially since the basic knowledge about specific effects and the knowledge about suitable survey and research instruments can still be extended. To this purpose, a set of key variables and dimensions has to be defined and sound instruments have to be developed.

On a theoretical level, normalization theory was a landmark in therapeutic architecture, as was the Safewards model for promoting safety in psychiatric care (39), but new models building on NT have acknowledged the limitations of deinstitutionalization and propose a synthesis of normalized and purposeful architectures and designs (37, 57). Moreover, the question remains as to what extent theories from therapeutic environments are applicable in high security environments (prison, forensic hospital). The impact of the physical environment on treatment, rehabilitation, safety and well-being in high-security environments needs further study.

In all research efforts in this field of work, much more attention must be paid to the cultural dependence of research approaches and results. Prison and forensic services are highly country-specific. Therefore, efforts have to be made to generate evidence-based knowledge on best practices in architecture and design in high security contexts which reflect cultural effects and diversity.

## Author Contributions

TR and MF conducted the literature search. TR wrote, reviewed, and supervised the manuscript. MF wrote, edited, and reviewed the manuscript. JB contributed to content aspects and reviewed the manuscript. All authors contributed to the article and approved the submitted version.

## Conflict of Interest

The authors declare that the research was conducted in the absence of any commercial or financial relationships that could be construed as a potential conflict of interest.

## Publisher’s Note

All claims expressed in this article are solely those of the authors and do not necessarily represent those of their affiliated organizations, or those of the publisher, the editors and the reviewers. Any product that may be evaluated in this article, or claim that may be made by its manufacturer, is not guaranteed or endorsed by the publisher.
