# Supplementary material for: Space and Well-Being in High Security Environments
Source: Front Psychiatry. 2022 May 30;13:894520. doi: 10.3389/fpsyt.2022.894520 (PMC9195501; doi:10.3389/fpsyt.2022.894520)
Supplement: Supplementary file 1 [file Data_Sheet_1.PDF]

Included studies

Table 1
Hospital and prison overcrowding

	First author (year)	Type of publication	Country of origin	Setting
1	Baggio et al. (2020)	Original study	Switzerland	Prison
2	Fazel et al. (2017)	Review	UK	Prison
3	Franklin et al. (2006)	Review	USA	Prison
4	Haney (2015)	Review	USA	Prison
5	Martin et al. (2012)	Original study	USA	Prison
6	Rabe (2012)	Original study	Germany	Prison
7	Van Ginneken et al. (2017)	Original study	UK	Prison
8	Virtanen et al. (2011)	Original study	Finland	Acute-care psychiatric in-patient hospital wards
9	Walker et al. (2013)	Review	UK	Prison
10	Wolff et al. (2015)	Original study	Switzerland	Prison

Table 2

Therapeutic architecture and design: Models and findings in non-forensic settings

	First author (year)	Type of publication	Country of origin	Setting
1	Aljunaidy et al (2021)	Review	Turkey	Miscellaneous
2	Backhaus (2008)	Original study	USA	Therapy room
3	Bowers (2014)	Theory/ Methods	UK	In-patient psychiatric ward
4	Carthey (2006)	Theory/ Methods	Australia	Health facility
5	Chrysikou (2012)	Theory/ Methods	UK	Mental health care facility
6	Conellan et al. (2013)	Review	Australia	Mental health care facility
7	Finch et al. (2022)	Review	UK	Psychiatric in-patient / forensic psychiatric wards
8	Noble et al. (2021)	Original study	USA	psychotherapy waiting room
9	Oostermeijer et al. (2021)	Review	Australia	Mental health facility
10	Sinclair (2021)	Original study	UK	Therapy room
11	Vischer (2009)	Theory/ Methods	Canada	Miscellaneous

Table 3

Architecture and design in high security environments: Prisons and forensic hospitals

	First author (year)	Type of publication	Country of origin	Setting
1	Beijersbergen et al (2016)	Original study	Netherlands	Pre-trial detention centre
2	Connellan et al. (2013)	Review	Australia	Mental health care facility
3	Degl’Innocenti et al. (2020)	Original study	Sweden	Forensic hospital
4	Eggert et al. (2014)	Original study	USA	High security forensic psychiatric facility
5	Grant et al. (2015)	Theory/ Methods	Australia	Prison
6	Morris et al. (2014)	Original study	USA	Prison
7	Olausson et al. (2021)	Original study	Sweden	Forensic hospital
8	Research and Evaluation Unit SPPS (2018)	Review	Sweden	Prison
9	Van der Schaaf et al. (2013)	Original study	Netherlands	Psychiatric and forensic wards
10	Wijk et al. (2019)	Original study	Sweden	Forensic hospital
